# The psychological concept of social sustainability in the workplace from the perspective of sustainable goals: A systematic review

**DOI:** 10.3389/fpsyg.2022.942204

**Published:** 2022-08-15

**Authors:** Darja Kobal Grum, Katarina Babnik

**Affiliations:** Department of Psychology, Faculty of Arts, University of Ljubljana, Ljubljana, Slovenia

**Keywords:** social sustainability, workplace, sustainable developmental goals, psychology, systematic review

## Abstract

Unlike environmental sustainability, social sustainability in the workplace is a relatively new concept that is still searching for its own definition and explanation. Therefore, in this paper, we systematically reviewed and critically evaluated recent research on this topic. In doing so, we identified important constructs that help us better define and understand the phenomenon of social sustainability in the workplace. We focused on articles from 2016 to 2022 with content related to three Sustainable Development Goals (SDGs), namely health and wellbeing (SDG-3), gender equality (SDG-5), and decent work (SDG-8). Given the harrowing events of the past 2 years, triggered by the COVID-19 pandemic and the global impact of the war in Ukraine, we also wanted to learn whether other categories, such as security (SDG-11) and peace (SDG-16), are embedded in the concept of social sustainability at work. The articles we studied were found through EBSCOhost, specifically in the Academic Search Complete, Business Source Premier, APA PsycInfo, SocINDEX with Full Text, and GreenFILE databases. We selected 67 articles and organized them according to the four levels of research and practice in work and organizational psychology. In reviewing the literature, we identified several constructs that can be classified at four levels of interest in work and organizational psychology. At the level focused on the job/work, we identified two main topics: (i) sustainable job/work characteristics and (ii) sustainable job (re)design. At the people-focused level, we identified the following topics: (i) pro-sustainable self-system, (ii) pro-sustainable job attitudes and motivation, (iii) sustainability work environment perceptions and other mediating mechanisms, and (iv) sustainable job behavior. The organization-focused level includes (i) organizations as human systems and (ii) pro-sustainable organizational policies and practices. The last (society-focused) level is defined by two main topics: (i) understanding society as a human system and (ii) pro-social mechanisms. In the discussion, we categorized specific constructs identified within the described focus levels into the theoretical model describing the psychological concept of social sustainability in the workplace from the perspective of sustainable goals.

## Introduction

The events of the last 2 years have dramatically advanced the threat to the concept of a sustainable society. The pandemic spread of the virus has thoroughly exhausted us, including psychologically (e.g., Frounfelker et al., 2022; Robinson et al., 2022; Solmi et al., 2022). In mid-February 2022, the pandemic finally ebbed somewhat, but on February 24, 2022, the world was inundated with news of the start of war between Russia and Ukraine. We were indirectly and directly involved in the war events, which caused significantly more worries and threats every day (APA, 2022). The first public opinion survey (Valicon, 2022) shows that the level of concern and pessimism (especially in Slovenia and Croatia) is higher than during the pandemic. Therefore, the concepts associated with the notion of sustainability seem like nice but once again distant wishes. Why is this so? The concepts like sustainability, sustainable development, social sustainability, etc. come from the core of humanity. Let us remember. In 1987, Norwegian politician Gro Harlem Brundtland (World Commission on Environment Development-WCED, 1987) introduced the concept of sustainable development, defining it as humanity's ability to “…meet the needs of present-day humanity without compromising the ability of future generations to meet their own needs” (World Commission on Environment Development-WCED, 1987, p. 16). This definition makes it clear that it is people who are at the center of creating and understanding the phenomenon of sustainability. It is we, the people, on whom the future of ourselves and our planet depends.

The authors of this article believe that this is precisely why it is important to explore and draw attention to the importance of sustainable development and sustainable society. We believe that in the ideas of sustainable development and social sustainability, it is possible to find the anchor points of human existence where people feel sufficiently secure and stable so that we, as individuals and as a society, pursue the goal of sustainability. One such anchor point is work and the sustainability it brings to the workplace. Blewitt defines sustainable development as “the idea that the future should be a better, healthier place than the present” (Blewitt, 2008, p. ix), and we connect this to the realm of work and believe that the future should include a “better, healthier workplace than the present.” Our main question can therefore be formulated as follows: How can social sustainability be developed in the workplace in the current turbulent times? Here we focus on the psychological dimensions of finding an answer. In what follows, we introduce the basic areas of sustainability, focusing on social sustainability. We then address the understanding of sustainability in the workplace (SSWP), and in the main section we develop a method for systematic review and interpretation of the results obtained.

Since Adams (2006) formulated the model of three interlocking circles of sustainability that are in balance with each other in his book “The future of sustainability: re-thinking environment and development in the twenty-first century,” this model has been used in numerous research projects on sustainability. Adams assumes three domains or intersecting circles of sustainability: environmental, economic, and social. Environmental sustainability refers to concern for the environment, rational use of natural resources and environmental management, and pollution prevention. Its goal is to find solutions that ensure that current interactions with the environment are carried out according to the principle of keeping the environment as natural as possible while constantly striving for ideal conditions. Economic sustainability includes concern for profit, business performance and growth. Its goal is to contribute to economic development, preservation, and creation of new jobs.

These two areas are quite well-defined and researched. However, the situation is different with social sustainability (SS), which still seems to be in search of its own definition and explanation. This is probably why “social” has been integrated late into sustainable development debates (Eizenberg and Jabareen, 2017). Different authors have defined SS differently, although their definitions agree on the point that SS is a cornerstone for understanding overall sustainability and sustainable development. Polese and Stren (2000), for example, define SS as “development that is compatible with the harmonious development of civil society and promotes an environment that fosters compatible coexistence among culturally and socially diverse groups while fostering social inclusion, with improvements in the quality of life for all segments of the population” (2000, p. 229). Colantonio (2010) defines it as a condition and process that improves the quality of life of a community. Other authors (Valdés-Vásquez and Klotz, 2013; Mostafa and El-Gohary, 2014) associate SS with the adequate distribution of quality of life in the present and in the future. Grum and Kobal Grum (2020) outline that “researchers agree that without socially oriented practices, sustainability efforts will be undermined because there are too many gaps in practice and theory” (Grum and Kobal Grum, 2020, p. 788).

Researchers also disagree on the structure of SS. Eizenberg and Jabareen (2017) recognize at least three components of SS: social capital, human capital, and quality of life. On this basis, sustainable social development meets all people's needs and leads to their satisfaction, happiness, security, health, and quality of life. Australian psychologists Magee et al. (2012) divide SS into four categories: economic, ecological, political, and cultural, again suggesting a slightly different understanding of SS. They divide each of these categories into three subcategories: confidence, concern, and optimism about the future. On this basis, they also developed the Social Sustainability Survey (Magee et al., 2012), which measures these four categories. Correlations with domains of wellbeing measured by the Australian Unity Wellbeing Index (Cummins et al., 2003) were found to be relatively high for all six domains: community satisfaction, environment, personal relationships, workplace, safety, and general satisfaction. In this way, life satisfaction was confirmed as an important component of SS (e.g., Eizenberg and Jabareen, 2017).

In 2015 (The Global Goals, 2022b), United Nations member states defined 17 Sustainable Development Goals (SDGs) and presented them in a General Assembly resolution. The plan is for us as a society to meet these goals by 2030. Among the goals are three that relate directly to SSWP (Contreras et al., 2022): SDG-3: Ensure healthy lives and promote wellbeing for all at all ages; SDG-5: Achieve gender equality and empower all women and girls; SDG-8: Promote sustained, inclusive, and sustainable economic growth, full and productive employment, and decent work for all. Based on their assumptions, the same three SDGs were utilized as the baseline for our scientific review.

Employee health and wellbeing, which relate to SDG-3, are critical to both the growth of the businesses in which employees work and to economic growth and development in general. The fact that wellbeing is closely linked to SSWP is also supported by the psychological research (Magee et al., 2012). When we place SDG-3 in the context of world events over the past 2 years, we see that researchers are addressing them more urgently. Specifically related to occupational health and wellbeing, much research was conducted during the COVID-19 pandemic that has helped to enrich our knowledge of the importance of occupational health and wellbeing (e.g., Kniffin et al., 2021; Shao et al., 2021; Vu et al., 2022).

SDG-5, which addresses gender equality, is becoming increasingly important in the workplace. Research clearly shows that there is no difference in work performance between men and women. As leaders, women can create a more positive work climate and show more empathy toward subordinates than their male counterparts (e.g., Regan et al., 2018; Saleem and Ajmal, 2018). Nevertheless, research also shows that as a society we are still far from full gender equality. On average, women are still paid less than men for the same work, with wages 10–30 percent lower than men, while men still predominate in leadership positions (Albuquerque et al., 2020; The Global Goals, 2022a). Discrimination against individuals with non-binary gender identities is even greater, and they are still at risk of losing their jobs or not getting a job at all if they disclose their gender identity. Research on this topic still lags, as there is relatively little published research on this topic compared to other vulnerable groups (e.g., Goldberg et al., 2021).

SDG-8 focuses on decent work, which is closely linked to SSWP and in this way intertwined with the goals related to wellbeing and gender equality mentioned earlier. Work environments, public, private, non-governmental, service and production organizations are the fundamental building blocks of sustainable development in society, as they both ensure the achievement of the SDGs in work environments where individuals spend most of their time in their active working lives and have a direct impact on the achievement of the SDGs in the broader society through their processes and structures. The psychological concept of decent work (Blustein et al., 2016; Duffy et al., 2016) at the individual level explains the role of decent work in a person's mental and physical health. As McWha-Hermann et al. (2021) note, a living wage is a key element of decent work and a decent life. However, it is primarily the complexity of the concepts that work and organizational psychology (WOP) deals with (individual, teams, organization, individual and organization in the broader social environment) that can pose a problem when studying SS. Indeed, the gap between micro and macro levels is one of the fundamental features of research on the social responsibility construct (Glavas, 2016). Some methods that are better suited for studying concepts directly related to social justice, such as intersectionality (Grzanka et al., 2020), are often less established in psychological science (Grzanka et al., 2020). For this very reason, the question of to what extent, with what focus, and how WOP can contribute to the SS of organizations and society in the future is a central question that we attempt to answer in this literature review.

**Figure 1:**
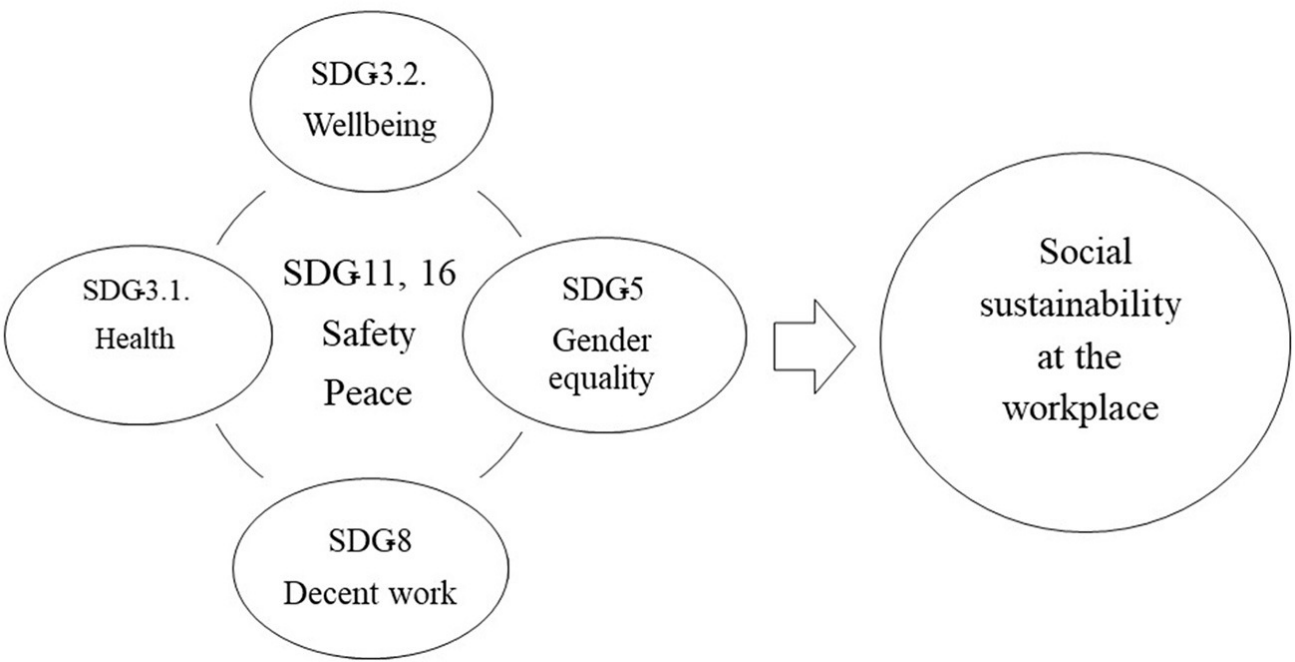
Hypothetical model of key sustainable development goals for social sustainability in the workplace at the start of the new decade.

In the last 2 years, the sense of worry, threat, and suffering related to the consequences of the pandemic COVID-19 and the war in Ukraine has greatly increased. As a result, people, especially Europeans, are also afraid of a general economic turnaround, job losses and poverty. The issues of peace and security are becoming increasingly important. For this reason, we have formulated a model of SS in the workplace based on the SDGs (Figure 1), which, considering the COVID-19 pandemic and the fear of war in Ukraine, we believe includes two additional SDGs that we need to examine very closely in the context of workplace sustainability in the coming decade. These are (The Global Goals, 2022b): SDG-11: Make cities and human settlements inclusive, safe, resilient, and sustainable, and SDG-16: Promote peaceful and inclusive societies for sustainable development, provide access to justice for all, and build effective, accountable, and inclusive institutions at all levels). So, there are two reasons for choosing the SDG-3, SDG-5, SDG-8, SDG-11 and SDG-16: a) the starting points set by Contreras et al. (2022) and the method of observing the global changes in the last 3 years with the emergence of COVID-19 and the war in Ukraine, which also affected the level of SSWP.

In summary, the insufficient involvement of psychological science in the study of multilevel constructs in SSWP (Blustein et al., 2016; Duffy et al., 2016; Grzanka et al., 2020) in the face of simultaneous global social situations that have changed and are changing existing concepts of work and life, has led us to the fundamental goal of this work to determine how SSWP is expressed through SDG-3, SDG-5, and SDG-8 and, given the emerging global situation, through SDG-11 and SDG-16. Figure 1 shows the theoretical framework for our study.

Based on the problem of the study and hypothetical model presented in Figure 1, we systematically reviewed the literature that addresses the SS concept in the context of the research and practice of WOP. The objectives of our study were the following:

- Identify key themes or constructs through which WOP contributes to understanding and ensuring SS.
- Identify what does the present mean for the advancement of the concept of SS and the role that psychology, particularly WOP, plays in it?
- To describe what role did or do the current conditions of pandemic and social insecurity play in describing and interpreting the factors and mechanisms for achieving the SDGs (SDG 3, 5, 8, 11, 16).

By reviewing the literature, we aimed to provide a general overview of the role of WOP in the study and application of SS concepts and to provide theoretical guidance for the further development of psychological science related to SS development and work.

## Materials and methods

### Search protocol

We conducted the literature search in accordance with the PRISMA protocol (Moher et al., 2009; Page et al., 2021). We searched for scholarly articles in the EBSCOhost databases, specifically Academic Search Complete, Business Source Premier, APA PsycInfo, SocINDEX with Full Text, and GreenFILE using the search term (with no restriction on where the words were found) “social sustainability AND work^*^.” The searches were conducted in March 2022.

Before determining the final search term, we familiarized ourselves with the results of several other search terms [“social sustainability”; “social sustainability AND business”; “social sustainability AND workplace”; “social sustainability and (work psychology OR industrial psychology OR organizational psychology,” “social sustainability AND psychology”)] that proved to be too broad (“social sustainability”; “social sustainability AND psychology”) or too narrow [“social sustainability and (work psychology OR industrial psychology OR organizational psychology”)] to provide insight into the concept of social sustainability in the context of WOP and the contribution of this psychological discipline to achieving the SS goals. A review of hits using the final search term “social sustainability AND work^*^” was confirmed to be stable, as hits obtained using narrower search terms [e.g., “social sustainability and (work psychology OR industrial psychology OR organizational psychology”)] were also obtained using the final search term used, while hits used as the basis for creating a literature review were also obtained using broader search terms (e.g., “social sustainability” AND “psychology”).

### Inclusion and exclusion criteria

The first search (with no search criteria at all) using the search term “social sustainability AND work^*^” yielded 3,753 works. In the next step, we narrowed the search using automated tools based on the following criteria:

Time: We covered the period of the last full 5 years, from January 2016 to February 2022, since 2016 was the publication year of the review article which focuses on the social responsibility construct and organizational psychology (Glavas, 2016). Moreover, as we noted in the introduction, current behavior, practices, norms, and values in society have been challenged in recent years due to the global crisis emanating from the COVID-19 pandemic.

- Type: works published in academic journals.
- Accessibility: fully accessible articles.
- Quality: peer-reviewed articles.
- Language: articles published in English.

### Data extraction

According to the automatically determined criteria, 279 works remained. Of these, the system removed 34 works as exact duplicates. What remained were 245 articles, which we can divide methodologically into review articles and original scientific articles reporting research conducted using quantitative or qualitative methods, and theoretical articles. In the second stage, we screened the 245 articles by title, abstract, and topic indicators. In the first stage of the screening process, we excluded 165 papers. The reasons for exclusion were as follows:

- Type of scientific article (editorials).
- Not relevant age group (not including working population).
- Exclusively dealing with economic or environmental aspects (energy sources, environmental analysis, ecosystems, environmental management), urban planning, aspects of public administration in communities, supply chain management.
- Too general theoretical works.
- Duplicates that the system did not automatically exclude.

**Figure 2:**
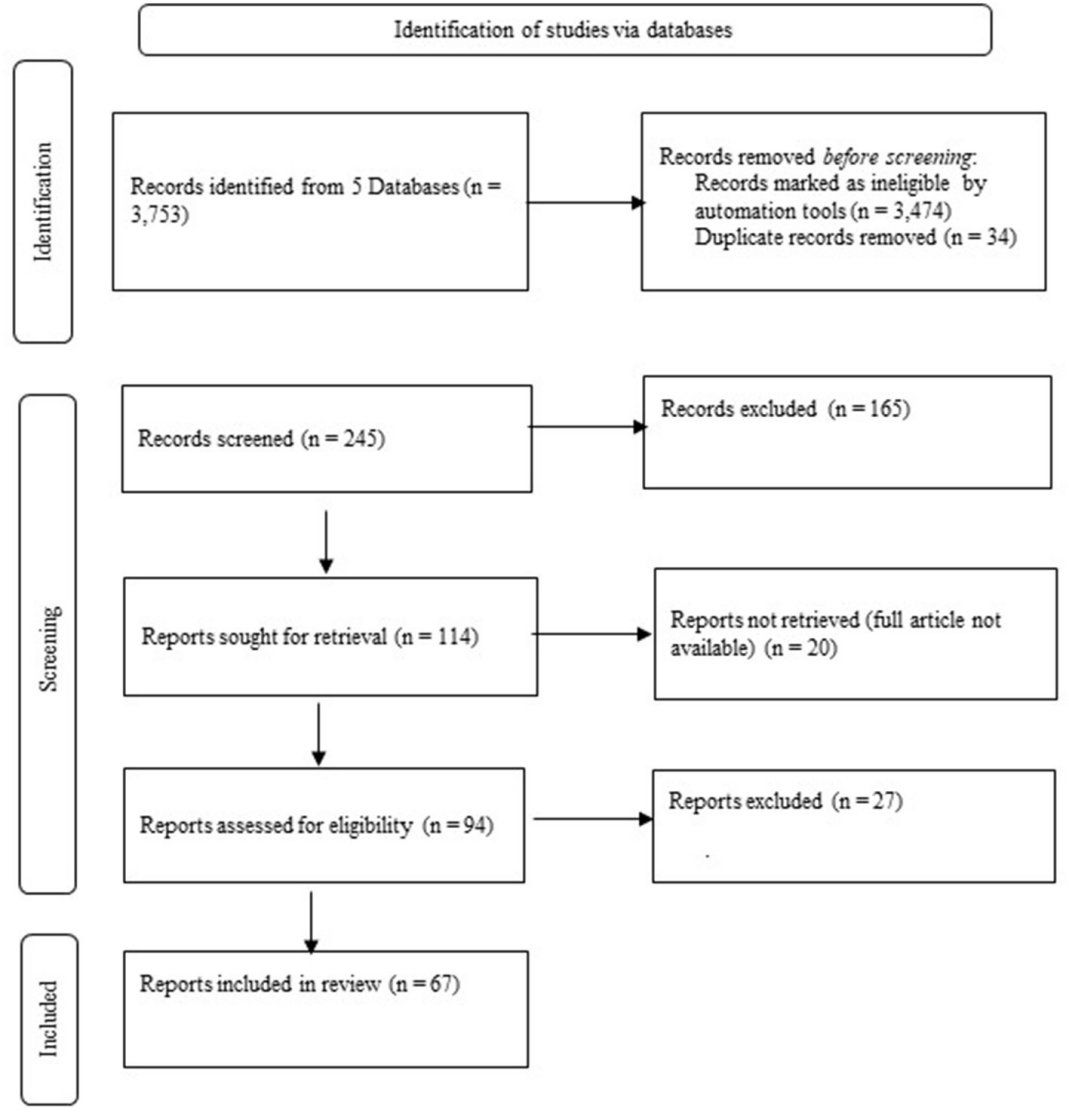
PRISMA diagram for the search protocol and the inclusion and exclusion of reviewed articles.

We attempted to obtain the remaining 114 articles in their entirety, but found that 20 articles were inaccessible, while among the other 94 articles were 27 whose content did not fit the objectives and research questions of the review. We therefore included 67 articles in the literature review, which are presented in the Results chapter (**Tables 2**–**5**). The entire process of the literature search is shown in the PRISMA diagram in Figure 2.

**Figure 3:**
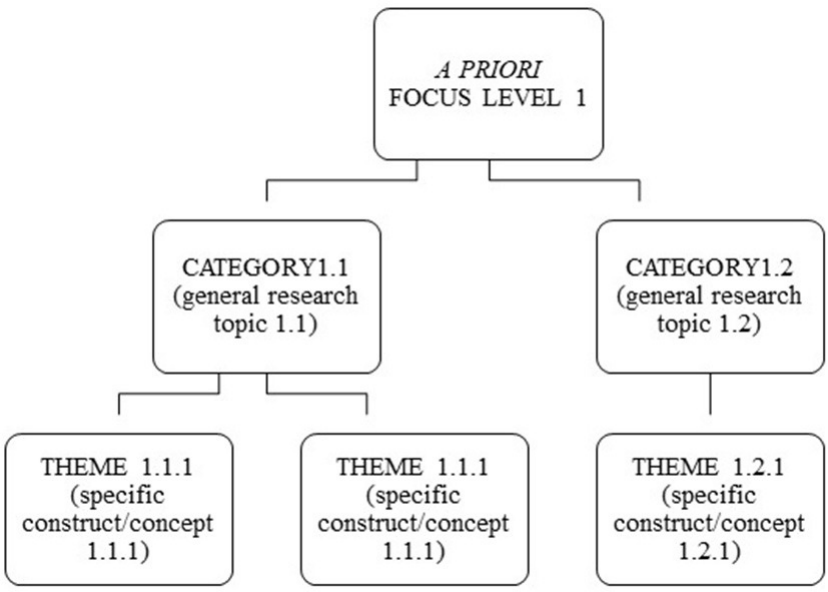
The process of classifying identified empirical constructs and theoretical concepts in a previously defined focus level of study of the work and organizational psychology.

We conducted the systematic review of the articles using a combination of deductive and inductive approaches. We started from the level of focus on the WOP as described in classical textbooks in Europe and the United States (Landy and Conte, 2013; Chmiel et al., 2017). Consistent with this, we defined four content levels of literature review, namely topics focused on work (job-focused), people at work and in work organizations (people-focused), the organization as a whole and its relationships within and outside the organization (organization-focused), and society (society-focused). The first three levels are consistent with the naming of the chapters in the (Chmiel et al., 2017) monograph, and the last one was added due to current issues in WOP (e.g., multiculturalism, Landy and Conte, 2013) and the role of WOP in society. Table 1 in the Results chapter shows the identified categories within each a priori defined focus level of WOP (work, people, organization, and society). We defined the first level of identified themes based on classical constructs that have been researched and applied in practice in WOP and that are also presented in traditional textbooks on WOP (e.g., Landy and Conte, 2013; Chmiel et al., 2017). We have called them categories because they represent general Research Topics in the study and interpretation of SS. The second level is formed by the “conceptual themes” (Vaismoradi et al., 2016, p. 104), i.e., the constructs we identify in relation to SS based on specific variables or theoretical concepts discussed in the identified articles. The review of the literature was therefore based on a priori levels of focus from WOP. We described the specific focus level based on empirical constructs and theoretical concepts we identified in the review of the literature. The identified empirical constructs or theoretical concepts were categorized into two levels, namely the categories (general Research Topics) and the specific constructs or themes (subordinate level) in relation to SS. Figure 3 shows the process of classifying the identified constructs and theoretical concepts.

**Table 1:** A priori focus levels of the review and identified categories (Research Topics).

**Focus level of research and practice in the WOP**	**Identified categories (Research Topics)**
Job/work-focused	Sustainable job/work characteristics
	Sustainable job (re)design
People-focused	Pro-sustainable self-system
	Pro-sustainable job attitudes and motivation
	Sustainability work environment perceptions and other mediating mechanisms
	Sustainable job behavior
Organization-focused	Organizations as human systems
	Pro-sustainable policies and practices
Society-focused	Societies as human systems
	Pro-social mechanisms

The authors conducted the entire process of reviewing the identified work in parallel, with intermediate coordination. This involved not only harmonizing the identified constructs and concept networks, but also interpreting them from the broader focus of psychological science, as the authors work in different psychological disciplines (personality psychology and WOP).

In the next chapter, the results of the literature review in accordance with the research questions are presented. The discussion includes a synthesis of the main findings from the literature review and a look into the future or a description of the future role of the WOP and the science of psychology in general in SS. The conclusions summarize all of this and point out the limitations of this literature review.

## Results

Table 1 presents the a priori defined focus areas of study and practice of WOP with the topics (categories) that we identified and named within each focus level according to the defined subordinate themes and constructs. Due to the volume of second-level constructs (themes) and citations, the themes for specific focus levels of WOP are presented in individual tables (Tables 2–**5**).

**Table 2:** Job/work-focused Research Topics and constructs.

**Research Topics identified in the review of the literature (categories)**	**Constructs identified in the review of the literature (themes)**	**Sources**
Sustainable job/work characteristics	Decent work Meaningful work Sustainable work	Benstead et al. (2018); Mushfiqur et al. (2018); Neumann et al. (2018); Guerci et al. (2019); Röös et al. (2019); Sun et al. (2019); Alexander et al. (2020); Forbes-Mewett et al. (2020); Sajjad and Shahbaz (2020); Trautrims et al. (2020); Conigliaro (2021); Duval et al. (2021); Harlin and Berglund (2021)
Sustainable job/work (re)design	Ergonomics Sustainable certification systems Innovation through new organizational processes and professional roles	Andriolo et al. (2016); Lake et al. (2016); Schiavo (2016); Neumann et al. (2018); Papadopoulos (2019); Röös et al. (2019); Alexander et al. (2020); Lombard and Viviers (2020); Međugorac et al. (2020); Duval et al. (2021); Harlin and Berglund (2021)

The review of the literature was guided by the basic focus of a multi-layered psychological discipline and thus covers four levels: the work, the individual, the organization, and the broader social system in which the work is performed and the organization functions. On the job/work level we have identified two main Research Topics: (i) sustainable job/work characteristics and (ii) sustainable job (re)design. At the people level, we identified four topics of study: (i) pro-sustainable self-system, (ii) pro-sustainable job attitudes and motivation, (iii) sustainability work environment perceptions and other mediating mechanisms, and (iv) sustainable job behavior. The level of organization includes two fields, (i) organizations as human systems, which bring together very different constructs from the social sciences that describe and interpret the organization through the primary perspective of the organization as a human system, and (ii) pro-sustainable organizational policies and practices, which bring together constructs related to the management of the organization and people that can contribute to SS. The final level, the level of society, is not a primary level of inquiry in the WOP but emphasizes the role of the broader social context in the functioning of organizations and individuals as their members, especially in today's world, so we have defined it a priori as an independent level of focus. This level, like the organizational level, is defined by two main topics: (i) understanding society as a human system and (ii) pro-social mechanisms. In contrast to the other a priori levels (job, people, organization), the constructs and concepts described and interpreted empirically or theoretically at the society level focus on SS as a construct that describes quality of life efforts through the prism of social mechanisms to ensure equity and fairness, without any obvious link to the other dimensions of sustainability. For this reason, we called the second domain of the study pro-social mechanisms at the level of society rather than pro-sustainability as we did at the level of organization, individual, and work.

### Job/work-focused fields of study and constructs

Table 2 shows the fields of study and defining constructs identified in the review of the literature, as well as the sources identified in the review of the literature that address the constructs at the level of work or job.

The first field of interest in WOP is work: its characteristics defined by job duties, tasks, responsibilities, and authority; work environment; work equipment and tools; social relationships at work; organization of work in terms of schedules, work hours, nature of employment, etc. The review of the literature revealed the following key constructs or topics relating to the characteristics of contemporary work in the context of the concept of SS: decent work, meaningful work, and sustainable work.

The issue of working conditions that ensure the basic dignity of individuals in the specific work environment or in the broader society, also compared to the concept of modern slavery (Benstead et al., 2018; Trautrims et al., 2020) and precarity (Forbes-Mewett et al., 2020), is one of the fundamental goals of SS, as defined in SDG-5. Conigliaro (2021) defines decent work as the main element of SS, as SS is a balance between “the right to pursue personal fulfillment and to be protected as a human being” (p. 142). In line with the definition of decent work, Conigliaro (2021, p. 148) defines five dimensions of decent work and their indicators: (i) inequality, (ii) work conditions, (iii) vulnerabilities, (iv) social protection, (v) resilience factors. Decent work is a general concept that refers to the right to work based on equality, fairness, decent working conditions and the satisfaction of individuals' needs, such as the need for meaningful work. The construct of work-family balance is also linked to the construct of decent work, as noted by Mushfiqur et al. (2018). Mushfiqur et al. (2018) define work-family balance as “the interface of work and family and the consequences of these two variables on commitment to work, job satisfaction, family roles and social related themes” (p. 870). A study conducted among female physicians in Nigeria showed that their workload, demanding work and work environment, unsupportive relationships within that environment, and specific expectations related to traditional gender roles reduce female physicians' ability to balance work and family, and thus their overall satisfaction (Mushfiqur et al., 2018).

Meaningful work refers to the character of work that combines an individual's efforts with benefits to others (e.g., Guerci et al., 2019; Röös et al., 2019; Sun et al., 2019; Sajjad and Shahbaz, 2020). Röös et al. (2019) investigated how sustainability assessment models contribute to the social status of livestock farmers in Sweden. Meaningful work was found to be one of the components of work characteristics (along with good financial status, comparable standard of living, stress management, reasonable working hours) associated with farmer wellbeing. Meaningful work is a mediating construct between the assessment of the harmony of an organization's social mission and the individual's concept of self, which is especially important for workers who chose to work in social enterprises (Sun et al., 2019). Working in an environment that emphasizes SS rather than just the economic component inherently increases workers' perceptions of the importance of work, which in turn promotes positive attitudes toward work (Guerci et al., 2019). Decent work and meaningful work are interrelated constructs. Decent work specifically includes the elements (indicators) of job security, decent working conditions, equality, social security, and ensuring personal development. All these elements of decent work represent sources of personal resilience to possible factors of the labor market and social systems over which the individual has no control (Mushfiqur et al., 2018; Conigliaro, 2021), thus making a lasting contribution to an individual's positive career. Meaningful work, on the other hand, is a concept associated in the reviewed literature with the characteristics of work and organizational context that convey to the worker that the goals and effects of the individual's work and the functioning of the organization are aligned with the overall social good (Sun et al., 2019).

The construct of sustainable work is linked to the construct of decent and meaningful work. For example, the adoption of agri-environmental practices had a significant impact on the work characteristics and working conditions of livestock farmers in France (Duval et al., 2021). Although improving working conditions was rarely the main motivation for farmers to adopt agri-environmental practices, they played an important role in improving the quality of working conditions. Sustainable work encompasses all three dimensions of sustainability: economic, environmental, and social (Harlin and Berglund, 2021). Harlin and Berglund conducted a longitudinal study of how new (start-up) companies address the challenges of work and ensuring SS. The goals of ensuring sustainable work were those that drove the new company to focus on innovative approaches to rapid decision making while ensuring decent work that was aligned with individual development and environmental sustainability. This insight leads directly to the next thematic set - sustainable work (re)design.

Specific certification schemes focused on assessing and recognizing the sustainable orientation of organizations are not necessarily a sufficient condition for ensuring the characteristics of work that we can describe with the construct of decent and sustainable work, as such approaches are mainly focused on compliance with labor law (Alexander et al., 2020). Such compliance is an important but insufficient measure to ensure all elements of decent and sustainable work. Similarly, it is probably not sufficient to implement ergonomic measures, although these are particularly important in industry, which is an environment with health risks due to work processes (Andriolo et al., 2016). Innovation in terms of new approaches to implementing basic processes, as demonstrated by practices in agriculture (Röös et al., 2019; Duval et al., 2021) and in start-up companies (Harlin and Berglund, 2021), can contribute to a more comprehensive approach in ensuring elements of SS in the workplace. Innovation can also enter companies and organizations in the form of new job descriptions and job roles. Professional roles are developed by people during their education as they acquire knowledge, skills, competencies, approaches to work, and attitudes toward work. Although this construct is closely related to the societal level (in **Table 5** Society-focused: Education), these roles, especially in today's world where education is more focused on the applicability of knowledge, play an important role in changing the characteristics of work by aligning occupational roles, values, views, and goals of work with all three dimensions of sustainability (Međugorac et al., 2020) and fostering sensitivity to individuals' local community (Lake et al., 2016).

At the level of work and work environment characteristics, the reviewed articles focus on SS through an interpretation of constructs such as decent work, meaningful work, and sustainable work, and through the identification of approaches to redesign work so that such work and work environment characteristics are also achieved (ergonomic solutions, implementation of certification systems that influence work and work environment characteristics, innovations), and on the role of educational institutions and the education system in setting the work standards that guarantee a sustainable orientation through the design of professional roles. The review also shows that the majority of the presented articles within the job/work focus interpret SS through the perspective of social rights and equality; some (e.g., Röös et al., 2019; Duval et al., 2021; Harlin and Berglund, 2021) take a broader approach to the construct of SS as “sustainable social relations” (Magee et al., 2012, p. 245), encompassing economic, environmental, cultural, and political relations as interconnected aspects of sustainability that cannot be reduced to a single dimension of sustainability.

### People-focused constructs

The next level of study is people (Chmiel et al., 2017). Within this level, we identified constructs through a review of the literature that we categorized into four categories (see Table 3): pro-sustainable self-system, pro-sustainable job attitudes and motivation, sustainability work environment perceptions and other mediating mechanisms, and sustainable job behavior.

**Table 3:** People-focused Research Topics and constructs.

**Research Topics identified in the review of the literature (categories)**	**Constructs identified in the review of the literature (themes)**	**Sources**
Pro-sustainable self-system	Personality Spirituality Environmental identity	Ciocirlan (2017); Kim et al. (2017); Wahid and Mustamil (2017); Sajjad and Shahbaz (2020); Anwar and Clauß (2021); Raniga (2021)
Pro-sustainable job attitudes and motivation	Trust Positive job attitudes Intrinsic motivation	Barin Cruz et al. (2016); Jitmaneeroj (2016); Carmeli et al. (2017); Ciocirlan (2017); Kim et al. (2017); Mäkiniemi and Heikkilä-Tammi (2018); Mushfiqur et al. (2018); Paillé et al. (2018); Salminen et al. (2018); Guerci et al. (2019); Rogge and Van Nijverseel (2019); Sun et al. (2019); DeMatthews and Izquierdo (2020); Farooq et al. (2020); Mariappanadar (2020); Međugorac et al. (2020); van Dick et al. (2020); Wynne-Jones et al. (2020); Harlin and Berglund (2021); Raniga (2021); Yin and Jamali (2021)
Sustainability work environment perceptions and other mediating mechanisms	Perceptions of organization's (social) sustainability Person-organization fit Organizational identification Perceived distributive justice Perceived support	Carmeli et al. (2017); Ciocirlan (2017); Grimes et al. (2018); Mushfiqur et al. (2018); Paillé et al. (2018); Röös et al. (2019); Sun et al. (2019); DeMatthews and Izquierdo (2020); Mariappanadar (2020); van Dick et al. (2020)
Sustainable job behavior	Identity work Workplace sustainability behavior	Carmeli et al. (2017); Ciocirlan (2017); Kim et al. (2017); Grimes et al. (2018); Paillé et al. (2018); Sun et al. (2019); Farooq et al. (2020); Mariappanadar (2020); Sajjad and Shahbaz (2020)

In relation to SS and work, current research examines the following constructs located in the individual's self-system: personality, particularly personality traits (Kim et al., 2017; Anwar and Clauß, 2021), which are associated with various aspects of SS, and also constructs that are more dynamic and dependent on context, as opposed to more stable personality traits, such as moral reflectiveness (Kim et al., 2017), self-awareness (Raniga, 2021), mindfulness and spirituality (Sajjad and Shahbaz, 2020), sense of calling (Wahid and Mustamil, 2017). Anwar and Clauß (2021) identified the important role of basic personality traits of business owners, except extroversion, in relation to the SS of businesses, through the individual's ability to effectively use the existing organizational resources (Anwar and Clauß, 2021). Conscientiousness and moral reflectiveness are associated with voluntary green behavior among employees and their managers (Kim et al., 2017). Sajjad and Shahbaz (2020) include mindfulness and spirituality among the psychological constructs associated with SS. At the individual level, the role of mindfulness is primarily to stimulate positive feelings, reduce negative emotional states and moods, and increase the ability to learn and solve problems. In a theoretical work, Ciocirlan (2017) describes environmental identity, which refers to the individual's conception of himself or herself as a being connected and attached to the natural environment, while the natural environment represents a value higher than humanity itself. A “sense of calling” (Wahid and Mustamil, 2017, p. 264) contributes to a balanced focus of organizations on people, nature, and economic value as it provides “meaning and purpose by making contributions to others” (p. 264).

More than the role of relatively stable individual characteristics in SS, the identified articles focus on examining the impact of various elements of sustainability on workers' attitudes, motivation, and behavior. At the heart of psychological science and its applied discipline WOP is the classic explanatory process of the role of perceptions (beliefs) and evaluations (attitudes) in behavior. In identified articles, the most often studied attitudes were job satisfaction, commitment, and work engagement. Another specific attitude that appears in the reviewed articles is trust, which we identified as a separate construct because of its “dual” role. Trust represents an attitude toward work that states that individuals feel safe to experiment and engage in various workplace behaviors, such as those related to the environment (Ciocirlan, 2017). At the same time, trust is also a general indicator of the quality of relationships and thus of life, representing a key component of social capital (e.g., Barin Cruz et al., 2016; Sun et al., 2019), while it is also an indicator of the ability of organizations to build trust in relationships with various stakeholders (Jitmaneeroj, 2016; Harlin and Berglund, 2021). Job satisfaction appears in the articles mainly as a component of the main constructs of positive functioning—wellbeing and quality of life (Rogge and Van Nijverseel, 2019). Commitment is the bond between the individual and the group or other higher system. In SS, the studies reviewed theoretically examine, describe, or interpret the importance of organizational commitment (Carmeli et al., 2017; Guerci et al., 2019; van Dick et al., 2020), co-workers' commitment (Paillé et al., 2018), community commitment (DeMatthews and Izquierdo, 2020), environment commitment (Ciocirlan, 2017), commitment to work and the profession (Mushfiqur et al., 2018), and family commitment (Mushfiqur et al., 2018). Commitment described in these studies refers to an intense emotional attachment to the object of consideration, as it is based on mechanisms of identification (Carmeli et al., 2017; van Dick et al., 2020). Intrinsic motivation appears as a studied psychological construct in only one reviewed paper (Farooq et al., 2020). Virtually all the reviewed articles at the person-centered level, intentionally or only indirectly mention constructs such as engagement and commitment that point to the individual's intrinsic motivation in relation to SS or sustainability. Individual choices and behaviors in line with the principles of sustainability or SS should therefore be independent of external incentives and based on intrinsic motivation reflected in engagement and commitment.

The topics included in the work environment perceptions and other mediating mechanisms category include constructs that are classic mediating variables between the “objective” aspects of the work environment and employees' attitudes and behavior. In addition to organizational identification (Carmeli et al., 2017; van Dick et al., 2020), they also examine as mediating variables the perceived fit between an organizational sustainability orientation and employees' personal value system (Ciocirlan, 2017; Grimes et al., 2018; Sun et al., 2019), perceived organization's ethics of care (Carmeli et al., 2017), perceived organizational support (Mariappanadar, 2020), and peer support (Paillé et al., 2018). Justice also appears as a potential mechanism of workers' interpretations on SS of work organizations, as Röös et al. (2019) found that comparing one's standard of living with the perceived standard of living of others is an important component of wellbeing. Important behavioral constructs that are exclusively dependent variables in the identified empirical and theoretical articles are identity work (Grimes et al., 2018) and sustainability work. Sustainability work summarizes constructs that denote employees' organization-related voluntary behaviors beyond their formal duties—organizational citizenship behavior (van Dick et al., 2020), participation in corporate voluntary programmes (Farooq et al., 2020), and environmentally friendly workplace behavior (Ciocirlan, 2017; Kim et al., 2017; Paillé et al., 2018). Identity work is a process by which people enact their personal values through their choices and work behaviors, and in this way externalize and create in the organization the underlying conditions for authentic engagement and enactment of the values of SS (Grimes et al., 2018). Identity work is a mechanism of externalization of personal values that takes place only under the conditions in which the personal values are consistent with the values of the organization. Because the responsibility for decision making in organizations is most often assigned only to management, the self-initiated externalization of values through decisions and behaviors is often limited only to them. For this reason, the empirical research reviewed most often examines organizations' perceptions of sustainability initiatives. The perceived sustainability of organizations through mechanisms of assessing the congruence of personal values and those of the organization, as well as identification with the organization, promote employees' sustainable behavior (Ciocirlan, 2017; Kim et al., 2017; Sun et al., 2019; van Dick et al., 2020) and provide persistence in employment in SS organizations (Sun et al., 2019). The mechanism described is fully confirmed by the research of van Dick et al. (2020), based on which they conclude that organizations' investments in corporate social responsibility, which support both the social and environmental dimensions of sustainability, have an impact on employee motivation and behavior only when employees' values are aligned with the principles and values of SS. In this process, the social influences of management and employees play multiple roles. Kim et al. (2017) confirmed a multilevel model that, in addition to personality traits, confirms the role of managers' green behavior (providing a resource for SS model learning) and green advocacy among colleagues in work groups on employees' green behavior (Kim et al., 2017).

Identified articles that address SS at the individual level shows that only some of them (Wahid and Mustamil, 2017; van Dick et al., 2020) address the concept of SS holistically, as a mutual intertwining of different dimensions of sustainability. Other articles focus mainly on the environmental dimension (e.g., Ciocirlan, 2017; Kim et al., 2017), which promotes a comprehensive sustainable orientation of individuals and organizations (Kim et al., 2017). SS is presented as one aspect of the broader sustainable orientation of organizations, to which employees contribute through their attitudes and behavior. We also found that a larger body of the articles reviewed addresses the role of attitudes toward work and work motivation in promoting and managing SS behavior in organizations (e.g., Carmeli et al., 2017; Ciocirlan, 2017; Paillé et al., 2018; Guerci et al., 2019; van Dick et al., 2020). Only to a lesser extent (such an approach is found in Mushfiqur et al., 2018; Raniga, 2021) do they address the impact that such behavior has on other areas of life and on workers' decent lives and general wellbeing. This suggests that the micro-level study focuses primarily on understanding the role of people as sources for achieving (social) sustainability, rather than on work organizations as sources and mechanisms for ensuring social justice and equity.

### Organization-focused constructs

We divided the review of articles studying the organization level into two categories, organizations as human systems and pro-sustainable policies and practices (Table 4). Within the category organizations as human systems we identified the topics organizational culture, organizational image, social capital, institutional commitment and engagement, and workplace diversity.

**Table 4:** Organization-focused Research Topics and constructs.

**Research Topics identified in the review of the literature (categories)**	**Constructs identified in the review of the literature (themes)**	**Sources**
Organizations as human systems	Organizational culture Organization's image Social capital Institutional commitment and engagement Workplace diversity	Appelbaum et al. (2016); Barin Cruz et al. (2016); Jitmaneeroj (2016); Vanderstukken et al. (2016); Carmeli et al. (2017); Ciocirlan (2017); Lima and de Oliveira (2017); Richardson et al. (2017); Wahid and Mustamil (2017); Gupta and Racherla (2018); Huang (2018); Mushfiqur et al. (2018); Paillé et al. (2018); Salminen et al. (2018); Khan et al. (2019); Sun et al. (2019); Alexander et al. (2020); DeMatthews and Izquierdo (2020); Gamble et al. (2020); Loor Alcívar et al. (2020); Sajjad and Shahbaz (2020); Wynne-Jones et al. (2020); Harlin and Berglund (2021); Pankaj and Seetharaman (2021); Raniga (2021); Yin and Jamali (2021)
Pro-sustainable policies and practices	Sustainable HRM Sustainable leadership Intra group relations and social influence Innovation Change management Ethics of care	Appelbaum et al. (2016); Vanderstukken et al. (2016); Carmeli et al. (2017); Ciocirlan (2017); Jansson et al. (2017); Kim et al. (2017); Mehta and Gorski (2017); Wahid and Mustamil (2017); Grimes et al. (2018); Mushfiqur et al. (2018); Paillé et al. (2018); Salminen et al. (2018); Williams (2018); Ashby et al. (2019); da Rosa et al. (2019); Guerci et al. (2019); Khan et al. (2019); McDermott et al. (2019); Röös et al. (2019); Bojner Horwitz et al. (2020); Charni (2020); DeMatthews and Izquierdo (2020); Ellinger et al. (2020); Farooq et al. (2020); Mariappanadar (2020); Sajjad and Shahbaz (2020); Wynne-Jones et al. (2020); Duval et al. (2021); Harlin and Berglund (2021); Raniga (2021); Yin and Jamali (2021); Devkota et al. (2022)

The construct of organizational culture, which has been one of the fundamental research foci of WOP in the last 20 years of the 20th century and the beginning of the 21st century, mostly does not appear directly as an object of study in the reviewed articles, but only indirectly as a construct that encompasses the set of values, norms, and behavior patterns that (co-)influence the work of individuals and organizations as a whole (e.g., Carmeli et al., 2017; Wahid and Mustamil, 2017; DeMatthews and Izquierdo, 2020) or specific organizational cultural traits that can promote organizational SS, such as the culture of exchange among organizational members that rewards cooperation among employees (Paillé et al., 2018) and promotes identification in the form of an “inclusive we-culture” (Harlin and Berglund, 2021), or a “network cooperations culture” resulting from partnership between organizations in the external environment (Barin Cruz et al., 2016, p. 1,005), and an environmentally friendly organizational culture (Ciocirlan, 2017). Organizational culture also appears as an aspect of the way organizations function in certain areas of activity, such as IT companies (Pankaj and Seetharaman, 2021). Loor Alcívar et al. (2020) define organizational culture as a component of organizational sustainability, or rather, they call it the “organizational identity,” which is composed of the “vision and mission, institutional values, and identification” (p. 326), and they include it as a new, fourth dimension of organizational sustainability (along with economic, social, and environmental) in the empirical model to analyze the relationships between corporate social responsibility (defined by the dimensions of economics, law, ethics, and philanthropy) and cooperative sustainability in Ecuador. Although by means of various structural models they confirm the predominant positive associations between the dimensions of sustainability and social responsibility, most of the variance is explained by the model that explains the SS of organizations through the dimensions of social responsibility. We therefore identified organizational culture theme not only because the construct appears in individual articles, but primarily because of the empirical work describing the relationships between the dimensions of sustainability in different samples of organizations (e.g., Jitmaneeroj, 2016; Gupta and Racherla, 2018; Loor Alcívar et al., 2020). In the case of tannery regions in India, Gupta and Racherla (2018) found a positive reciprocal relationship between economic and environmental success, but a negative reciprocal relationship between the social and economic success of tanneries. As Jitmaneeroj (2016) stated, the relationship between different pillars or dimensions of sustainability moderates the activities of organizations (the industry), and “each pillar has unequal effects on the overall corporate sustainability and that the overall score is affected by not only the direct effects from pillar scores but also the indirect effects from the causal interrelations among pillars.” (p. 1,497). Other identified articles also point to the contradiction between effectiveness and solidarity (Lima and de Oliveira, 2017), the balance between an organization's socioeconomic mission and exclusively economic goals (Gamble et al., 2020), and the balance between the principles of New Managerialism and Confucian ethics among teachers (Huang, 2018).

Other constructs that appear in the identified scholarly articles include organizational image (Vanderstukken et al., 2016); social capital (Barin Cruz et al., 2016; Sun et al., 2019; Alexander et al., 2020; Sajjad and Shahbaz, 2020; Raniga, 2021); institutional commitment (Barin Cruz et al., 2016; Alexander et al., 2020; DeMatthews and Izquierdo, 2020; Yin and Jamali, 2021); and workplace diversity (Mushfiqur et al., 2018; Khan et al., 2019). Workplace diversity is a key issue in pursuit of SDG-8 (decent work and economic growth) and SDG-5 (gender equality). In addition to the frequently discussed issue of gender equality in the labor market and in working and personal lives (Mushfiqur et al., 2018), there are still many barriers to labor market access and employment for people with disabilities when it comes to inclusion (Khan et al., 2019). Retention of older workers is also not a common practice in companies, especially in sectors that can themselves contribute to the extended working capacity of workers, even after meeting retirement requirements (Salminen et al., 2018). Social capital, together with human capital (knowledge, skills, values of individuals), is a defining element of corporate social responsibility (Alexander et al., 2020; Sajjad and Shahbaz, 2020), which defines the productive and trust-based exchange of the organization with the external environment, and in this way, the role of the organization's commitment or propensity to collaborate with different actors or partners (Barin Cruz et al., 2016; Yin and Jamali, 2021), with the local community (Alexander et al., 2020; DeMatthews and Izquierdo, 2020), and the broader society (Sajjad and Shahbaz, 2020) is emphasized. Organizational image, expressed in concepts of respectable organizations (the image of the organization as SS) and impressive organizations (commercially highly successful and prestigious organizations), attracts a variety of applicants for employment, depending on whether the applicants are primarily seeking fulfillment of intrinsic or extrinsic values through employment (Vanderstukken et al., 2016).

The category of pro-sustainable policies and practices is defined by sustainable human resource management, innovation, sustainable leadership, organizational ethics of care, intragroup relations and social influence, participation, and voice, change management. Sustainable human resource management (HRM) (Mariappanadar, 2020) emphasizes the principles of an organization achieving economic success through HRM that ensure employee involvement and motivation (e.g., career development, performance management, employee benefits), in addition to minimizing potential harms that such practices have on employees by limiting the amount of time employees spend on their health. Sustainable HRM is defined as an HRM system focused on developing human capital in organizations to achieve not only economic but also social and environmental effects (Salminen et al., 2018). HRM system focused on ensuring employee engagement and motivation may have negative effects on managing health-related risks (Mariappanadar, 2020). On the other hand, such system represents an important element in older workers' decision to stay in the workforce until retirement or longer (Salminen et al., 2018). In addition to HRM systems, the identified articles focus on individual practices and their role in the SS of organizations, such as the effect of employee training on connecting and ensuring SS local self-government (da Rosa et al., 2019), attracting employees through a value system consistent with SS principles (Vanderstukken et al., 2016; Ciocirlan, 2017), employing vulnerable groups (Khan et al., 2019; Ellinger et al., 2020), career orientation to SS activities and organizations (Mehta and Gorski, 2017), and occupational health (Röös et al., 2019). In addition to HRM, an important factor in promoting SS is leadership. More than specific leadership styles (e.g., spiritual leadership—Wahid and Mustamil, 2017), sustainable leadership is a construct that summarizes leaders' decisions, behavior, and communications (Ciocirlan, 2017; McDermott et al., 2019; DeMatthews and Izquierdo, 2020), as important learning models for employees (Kim et al., 2017). Leadership is only one of the forms of social influence in organizations that flows “top-down.” Changes toward voluntary employee participation in sustainable initiatives, such as green behavior, cannot be ensured without horizontal influences among employees in the form of support (Paillé et al., 2018) and altruistic behaviors, such as knowledge sharing among employees (Ciocirlan, 2017).

In the context of organizational policy and practice, innovative approaches and social innovations are an important element for the development of SS in organizations. For example, Ellinger et al. (2020, p. 339) describe the modern “blue ocean strategy” approach to proactive recruitment and inclusion of workers with disabilities, which, while a social innovation, can play an important role in changing the internal organizational environment. Innovations also include the implementation of volunteer programmes within the organization in which employees participate (Farooq et al., 2020). Innovation is in the core of fast-growing start-up companies whose fundamental management model is based on intensive management of change (Harlin and Berglund, 2021). Appelbaum et al. (2016) point out that developing sustainable organizations is a process of organizational change that often fails. This is likely in part because the primary principle of sustainability and SS of change must be an ethic of care (Carmeli et al., 2017; Williams, 2018), especially in a social environment facing a crisis of care and a financial, environmental, and social crisis. The ethical principles of care focus on people's needs, relationships, and the ethics and morality of decision-making (Carmeli et al., 2017).

The level of organization as the focus of WOP points to different but related concepts that link organizational culture, leadership, and practices to sustainability development. At this level of focus, the prevailing assumption is that sustainability is a construct that can only be addressed as a whole, with an orientation to all pillars or dimensions of sustainability. Specific SDGs can therefore be achieved through simultaneous efforts in different areas of organizational activity (social relations, attitudes toward the natural environment, financial operations). Similarly, constructs found at the macro level of society.

### Society-focused constructs

The identified topics in articles studying and interpreting SS at the societal level to a large extent reflect the topics at the level of the organization as a system of people (Table 5).

**Table 5:** Society-focused Research Topics and constructs.

**Research Topics identified in the review of the literature (categories)**	**Constructs identified in the review of the literature (themes)**	**Sources**
Societies as human systems	Societal culture Gender equality Vulnerable groups Human and social capital Decent life	Foy Connor and Bent-Goodley (2016); Mohapi (2016); Lima and de Oliveira (2017); Leinonen et al. (2018); Mushfiqur et al. (2018); Puga and Soto (2018); Zuev (2018); Ashby et al. (2019); Rogge and Van Nijverseel (2019); Röös et al. (2019); Aksoy and Arli (2020); Ballet et al. (2020); Ellinger et al. (2020); Forbes-Mewett et al. (2020); Sajjad and Shahbaz (2020); Conigliaro (2021); Ibrahim (2021); Raniga (2021)
Pro-social mechanisms	Social contract Politics Legislation Educational system and practices Crisis management Innovation Social partnership and voice Care-ethical approach	Foy Connor and Bent-Goodley (2016); Schiavo (2016); Lima and de Oliveira (2017); Benstead et al. (2018); Williams (2018); Zuev (2018); Khan et al. (2019); McDermott et al. (2019); Papadopoulos (2019); Pye (2019); Charni (2020); Lombard and Viviers (2020); Međugorac et al. (2020); Novitz (2020); Trautrims et al. (2020); Conigliaro (2021); Ibrahim (2021); Raniga (2021)

The societies as human systems category includes societal culture, gender equality, vulnerable groups, human and social capital, decent life, and wellbeing. At the societal level, individual articles focus on the characteristics of specific social environments in which the authors examine variables or processes. Predominant are studies that address cases at the level of developing countries and countries that face difficulties in securing SS and sustainability (Foy Connor and Bent-Goodley, 2016; Lima and de Oliveira, 2017; Mushfiqur et al., 2018; Ibrahim, 2021; Raniga, 2021). Like organizational culture, the dynamics between different dimensions of sustainability are also studied at the societal level. Based on the SDGs of UN and the “Happy Planet” index (Aksoy and Arli, 2020, p. 387), Aksoy and Arli (2020) conducted an analysis of the relationship between the sub-dimensions of sustainability of specific societies and the “happiness” index. The authors find that 94% of the variability (p. 388) of happiness at the societal level is explained by sustainability indicators, with the environmental and societal dimensions of SS positively associated with happiness, while the economic dimension is not associated with the societal happiness index. Gender equality and vulnerable groups are issues that appear in most of the identified items at the societal level of the study. Gender equality is one of the fundamental components of the concepts at the heart of SS—social justice, security, and cohesion (Ballet et al., 2020). Raniga (2021) notes that neoliberal social policies contribute significantly to the “feminization of poverty” (p. 592), as it is more difficult for women to break out of the vicious cycle into which they are pushed by systemic discrimination in the labor market, education, and other areas (Raniga, 2021). For other vulnerable groups modern society also does not provide a way out of the vicious cycle of insecurity and exploitation (Mohapi, 2016). Human and social capital are concepts that define positive individual and social power achieved through skills and solid social networks that are resources for development. As Puga and Soto (2018) found, only certain forms of social capital are important for labor market participation. These are social networks that include individuals with higher social status. Access to such networks is not available to all, especially to women (Puga and Soto, 2018). Based on a selection of specific indicators of the dimensions of decent work already described, Conigliaro (2021) established the degree of fulfillment of various indicators and the overall degree of decent for EU countries. Just as we can define the level of decent work at the societal level, we can also define the level of happiness (Aksoy and Arli, 2020) and quality of life (Rogge and Van Nijverseel, 2019) at the societal level. Here, the concepts of decent work and wellbeing form the common overarching theme of decent living. However, different classifications of countries in terms of achieving SDGs do not mean much if scholars do not simultaneously examine the mechanisms that can lead to change. We have grouped these into a main area of interest, which we call pro-social mechanisms.

Crisis situations, such as the sudden outbreak of the COVID-19 pandemic, can increase risks in ensuring decent work and lives (Trautrims et al., 2020). The COVID-19 pandemic resulted in extreme changes in demand patterns, with temporary production stoppages and border closures blocking supply chains and reducing the effectiveness of risk management, while increasing workers' vulnerability to exploitation throughout the supply chain (Trautrims et al., 2020). Companies' struggle to survive distracted them from social and environmental issues and reduced the effectiveness of mechanisms that prevent worker exploitation (Trautrims et al., 2020). The functioning of organizations inside and outside their boundaries was redirected to the dimension of operating organizations to ensure their economic survival. Organizations that maintain trusting relationships with their stakeholders and have strong relationships within their scope of operations have an advantage in crisis situations, as such elements of stakeholder relationship quality are an important factor in operational resilience (Trautrims et al., 2020). A crisis can therefore threaten the key mechanisms that can support the SS of organizations-particularly the mechanisms of control over operations and functioning in terms of worker protection. The pro-social elements of the social environment that increase (or decrease) risks to social justice and equality and to decent living are economic conditions and policies (Ibrahim, 2021), legislation (Benstead et al., 2018; Khan et al., 2019; Raniga, 2021), the education system, and labor market policies (Schiavo, 2016; Papadopoulos, 2019; Međugorac et al., 2020), while social innovations in various forms adapted to situations and target groups play an important role (Lima and de Oliveira, 2017; Benstead et al., 2018). The mutual influence of all the mechanisms is most evident in efforts to change the social contract, where attempts have been made to achieve social change through revolution (Ibrahim, 2021). A revolution involves exposing oneself to threats to one's security and social peace to improve the rights and welfare of citizens. Nevertheless, they do not necessarily achieve their goals, especially when political interests outweigh the interest in securing the fundamental rights of citizens to live in dignity. In such a society, disadvantaged (vulnerable) groups become even more deprived and vulnerable to poverty and unemployment (Ibrahim, 2021).

Social subsystems in a particular area, such as work and related social rights (Novitz, 2020) or the entire social system (Ibrahim, 2021), ensure SS only if they are based on a social contract that makes it possible to address and consider people's expectations on the one hand and the state's or community's responsibility for these expectations on the other. The very concept of social contract emphasizes participation and negotiation to achieve a balance of expectations and obligations for both partners (Ibrahim, 2021). International labor standards and social regulations represent the obligations of society and organizations to workers, but these mechanisms alone are not sufficient. Negotiations that imply participation, partnership, and voice (Novitz, 2020) are prerequisites for decent work standards to be met. Particularly in developing countries, disregard for workers' rights and lack of financial and professional incentives drive many workers into migration. The crises we have experienced in the recent past, such as the financial crisis and the subsequent intensification of austerity policies, the environmental crisis due to the exploitation of the world's natural resources, the crisis of devaluation of health services and social care, and the crisis of migration, threaten the security, solidarity, and sustainability of humanity (Williams, 2018). But these are crises that transcend narrow economic frameworks, so there is little interest in solving them. Coordinated implementation of social protection, labor and employment, and migration policies is needed, as well as positioning care (for self, for others; as policy, practice, service, or relationship) as a universal human practice and ethic (Williams, 2018). Although caregiving relationships can be inherently unequal, in relationships (between caregivers and care recipients) based on mutual responsibility, respect, and support, the giving and receiving of care are linked to trust, tolerance, and respect for diversity (Williams, 2018). Such care has cumulative (including economic) value: “The more people are supported, the better they are able to provide care” (Williams, 2018, p. 557).

## Discussion and conclusions

**Figure 4:**
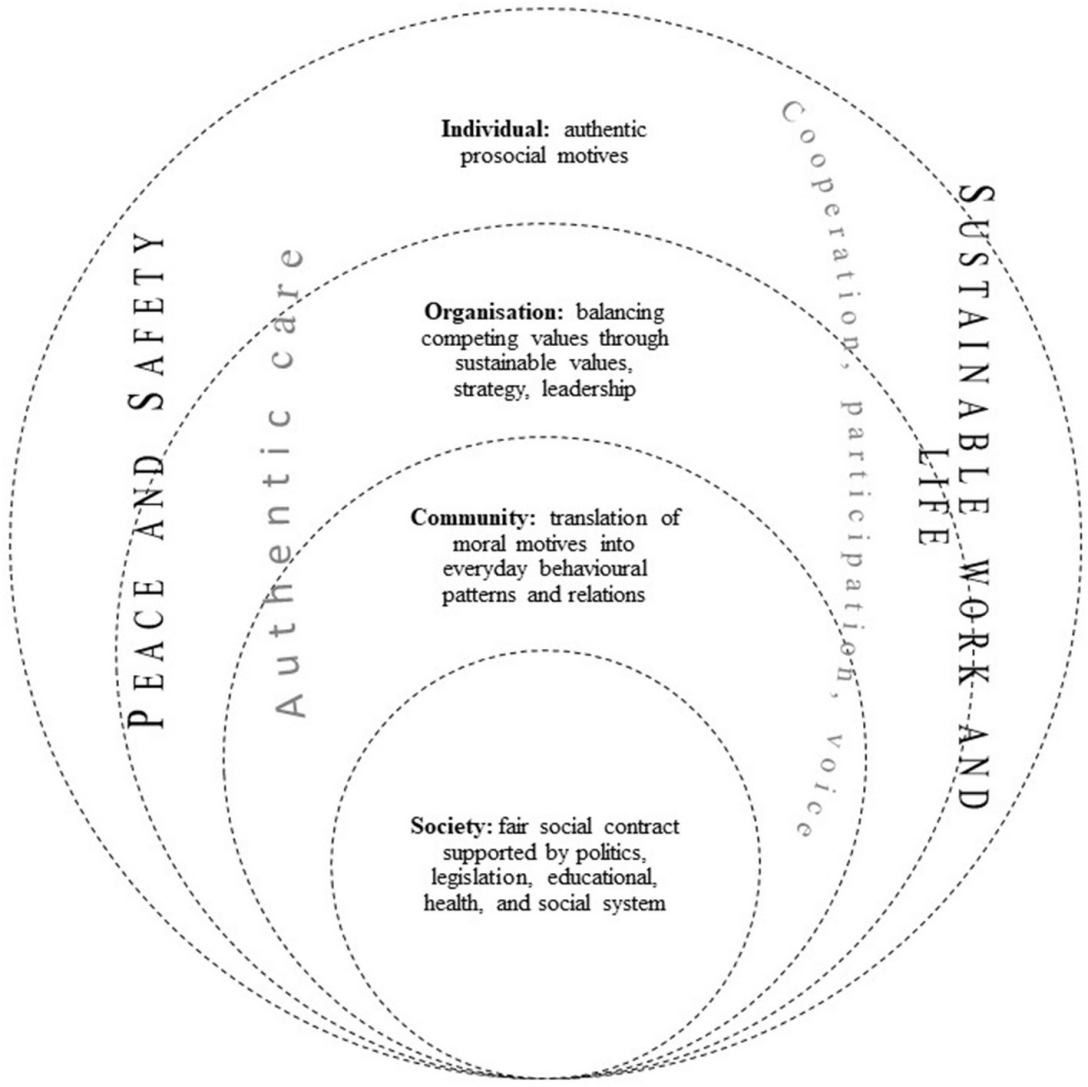
Theoretical model—psychological concept of social sustainability in the workplace from the perspective of sustainable goals.

The review of the literature was guided by the following three objectives: identification the key themes or constructs through which WOP contributes to understanding and ensuring SS; to identify of what does the present mean for the advancement of the concept of SS and the role that psychology, particularly WOP, plays in it; to find out what role did or do the current conditions of pandemic and social insecurity play in describing and interpreting the factors and mechanisms for achieving the SDGs. We presented the first, the identification of key constructs through which WOP contributes to understanding and ensuring SS in the present, in the Results chapter. Building on this, we have developed a comprehensive theoretical model of key constructs and mechanisms for promoting SS that simultaneously offers answers to the second goal set: What does the present mean for the advancement of the concept of SS and the role that WOP plays in it. In this chapter, we will bring the identified constructs together in a conceptual network to present the interconnectedness of the identified constructs and the SDGs and sustainability dimensions, focusing on the broader role of psychology and the WOP in ensuring SS. The major constructs or themes identified in the review of the literature are presented below and are shown merged in Figure 4.

Although some authors (e.g., Pappas and Pappas, 2015) have previously highlighted the importance of specific personality constructs that may denote an individual self-system, a review of the literature did not identify much work examining the role of individual traits in relation to SS behavior. Among the identified works are studies on the role of conscientiousness and moral reflexivity in sustainable behavior (Kim et al., 2017), the role of the five major dimensions of personality (Anwar and Clauß, 2021), intrinsic values (Vanderstukken et al., 2016), intrinsic motivation (Farooq et al., 2020), and mindfulness (Sajjad and Shahbaz, 2020). Although mindfulness provides a linking mechanism between the individual, the organization, and society (Sajjad and Shahbaz, 2020), as it allows one to step beyond the boundaries of the individual into relationships with the immediate and broader environment, further examination of the individual determinants of SS decisions and behavior must be mindful of the danger of redirecting SS-related concepts into individualistic, self-oriented need satisfaction. Stanley (2012) states that the study and application of mindfulness is based on the “inherent goodness of individual autonomy and responsibility, self-expression, personal development, enhanced subjective wellbeing, emotion regulation, and the pursuit of happiness irrespective of social conditions or ethical/moral conduct” (p. 632). Mindfulness training confirms the positive effects on wellbeing and quality of work (Mäkiniemi and Heikkilä-Tammi, 2018), but the conclusion that such an individual-focused approach promotes SS and sustainability may be incorrect or incomplete. This is because it implies the accumulation of wealth at the collective level without considering the dynamics within and between groups and the system or systems, which are not necessarily focused on social justice in the sense of prosperity for all, but on “exclusive” prosperity that depends directly on the social and economic status of the individual (Puga and Soto, 2018) or the collective. Sustainability can only be based on an ethic of life and work and a morality oriented to the common good that, more than utilitarian and instrumental values, emphasizes the relational component and the evaluation of decisions and actions in the context of their impact on others, not only “here and now” but through the perspective of the future—the sustainable impact. It is precisely this relational perspective that is an important unifying element for various micro-level (individual-level) dimensions of sustainability. Kim et al. (2017) underpin the importance of moral motives for sensitivity to social and environmental issues. Fundamental respect for human dignity determines individuals' attitudes toward people and the environment in terms of pro-social and pro-environmental attitudes and behavior (Kim et al., 2017).

Decent work is a construct that is central to all the articles presented, whether at the level of the characteristics of the workplace or at the level of society that seeks to provide a decent life for all residents, especially vulnerable groups, through decent work, despite possible disabling policies and social subsystems. The systematic review did not identify any article examining the psychological concept of decent work by Blustein et al. (2016) and Duffy et al. (2016). Drawing on well-founded criticisms of overly “Western” psychological approaches that focus on the core of individual wellbeing in relation to work constructs with a focus on personal agency beliefs and volition, which has greater interpretive power for a particular segment of the working population, Duffy et al. (2016) developed a multi-layered model or the new Psychology of Working Theory (PWT). This describes and explains the predictive factors, mechanisms, and consequences of decent work. Predictors of decent work include Duffy et al. (2016) psychological (e.g., work, career adaptability, proactive personality, social support), economic, and social (e.g., economic conditions, economic barriers, marginalization) variables that have been largely ignored in previous models of WOP. Decent work enables people to meet their needs, self-actualization at work, and wellbeing. Operationalization of the constructs described (Duffy et al., 2019a) and empirical research (Douglass et al., 2017; Duffy et al., 2019b) support the model. Review of the literature shows that the constructs we obtained in the literature review are included in the model PWT, for example, under the factors of personality or self-system, social support, vulnerable groups, economic conditions, and meaningful work. Further efforts to explain the role of WOP in SS should therefore be linked to PWT as the main explanatory model.

Sustainable work is a construct we identified in our literature review, and it describes work that encompasses all three dimensions of sustainability: economic, environmental, and social (Harlin and Berglund, 2021). Thus, it includes the dimensions of decent work, productive work, and environmentally sustainable work. Further research steps should be to improve the conceptualization, operationalization, and validation of the construct of sustainable work as a three-dimensional construct. Because previous research has well-defined the constructs of decent work and productive work and their interrelationship (Ford et al., 2011; Cerasoli et al., 2016), future research efforts should focus on the environmental dimension of work, e.g., objective and perceived environmental factors related to work, environmentally sustainable work behavior, and positive work-related outcomes related to the preservation of the natural environment.

Quinn and Rohrbaugh's (1983) competing values model of organizational culture summarizes the results of the literature review in the construct of organizational and social culture. As we have noted, the works identified do not directly relate to the elements or characteristics of organizational and societal culture as explained by WOP but rely primarily on identifying the reciprocal relationships between dimensions of sustainability and their predictive power, while individual works also directly describe the paradox of modern society and approaches in which individual professional groups balance economic and societal needs. The basic concept of the competing values framework (Quinn and Rohrbaugh, 1983) is managing the paradox of efficiency—balancing business outcomes, ensuring internal stability of operations, openness of the organization to the outside world and to innovation, and ensuring supportive practices and relationships within the organization. Managing (seemingly) paradoxical demands is a key responsibility of leaders and managers, who for this reason play a key role in developing culture and in ensuring organizational sustainability. This includes the identified construct of sustainable leadership, which, however, as the review of the literature shows, more than a specific leadership style, describes a sincere commitment of the leadership of organizations to achieve the goals of SS and to act according to the principles of SS, which is evident in the behavior and decisions of leaders (Ciocirlan, 2017; McDermott et al., 2019; DeMatthews and Izquierdo, 2020).

The review of the literature has shown that we should not separate SS from the other dimensions of sustainability. Each sustainability dimension contains elements of all three dimensions. SS may indeed be primarily focused on the positive social outcomes of health, wellbeing, poverty prevention, and decent living. However, the decent living component cannot be achieved in modern society unless all three dimensions of sustainability are addressed simultaneously. Further steps in the development of WOP to support the SS of organizations could focus on a theoretical upgrading and operationalization of the competing values model, which is derived from the basic dimensions of sustainability and enables an analysis of the situation at the level of organizations and their members in terms of the organization's alignment with all three dimensions of sustainability simultaneously.

Over the past5years, we have also noted a lack of research that seeks to explain not only how to promote various organization-friendly behaviors among employees (e.g., organizational citizenship behavior or sustainable behavior), but also would examine the role of the organization as an agent of change in the actions of individuals in other areas of their lives. An example might be the spread of green behavior from the work environment to the home environment of employees, or the role of the organization's social justice and inclusion efforts, as well as employee sensitivity to social issues. Although the identified research also emphasizes that organizations with a particular image attract candidates with different value systems (Vanderstukken et al., 2016), organizational socialization can still play an important role in raising employees' awareness of various SS issues. Although research (e.g., Cooper-Thomas et al., 2004) does not provide consistent confirmation of changes in employee value systems as a result of organizational socialization, it does confirm changes in the degree of perceived fit between individual and organizational values. The perceived fit of the individual's value system and that of the organization may indicate an adoption (though not necessarily an assimilation or identification) of decisions, principles, values, and value systems and, as such, may have a potential influence on the individual's decisions and behavior in other life contexts. At the individual level, which is the focus of WOP, it would be useful for future research to focus on the transfer of SS principles and behaviors from the organizational setting to other areas of life.

At the levels of the workplace, the organization, and society, the review of the literature identified numerous practices that ensure decent work and the SS of organizations and society. Among them, the construct of innovation, i.e., introducing new but proven policies and practices to ensure decent work, engaging vulnerable groups, improvement of operations and minimizing the negative impacts of operations on the natural environment, or all these elements simultaneously (e.g., Ellinger et al., 2020; Raniga, 2021), emerges repeatedly at all three levels. However, implementing innovative approaches requires support from societal-level policies and legislation, as well as organizational policies and practices, even though the innovations might (or even should) initially be conceptualized as countering existing cultural norms (Raniga, 2021). The concept of sustainable HRM can be an important contribution of WOP to the advancement of SS, especially through a more precise definition of the construct and empirical research on the long-term impact on employees and the operation of organizations.

In reviewing the identified articles, both at the level of the organization and society, constructs such as participation, cooperation, partnership, voice (Mushfiqur et al., 2018; Novitz, 2020; Yin and Jamali, 2021), as well as commitment, engagement (Carmeli et al., 2017; Ciocirlan, 2017; Mushfiqur et al., 2018; Paillé et al., 2018; Guerci et al., 2019; DeMatthews and Izquierdo, 2020; van Dick et al., 2020), and care (Carmeli et al., 2017; Williams, 2018) were described. These constructs embrace principles of relationships, processes, and work that involve all interested stakeholders in the internal and external environments of organizations, while drawing attention to the fact that the authenticity of these dynamics can only be achieved through an emotional connection (and thus an identification with stakeholder needs) and an authentic concern for all stakeholders in the organization. As such, they represent a specific *modus operandi* in systemic efforts to implement and achieve SS. They are relational by nature, as they describe the quality of organizations' relationships with individual members of the organization, with the organization's stakeholders, and with the broader society. The danger in highlighting such operating principles is that they may be trivialized as “social stuff” (Alexander et al., 2020) or associated with so-called New Age constructs (Farias and Granqvist, 2007), which may have a mimetic effect on the true meaning of supportive and collaborative approaches. For this reason, it is even more important that all social subsystems, especially those whose primary task is to educate future generations, be oriented toward linking dimensions of sustainability and toward the characteristics of individuals that ensure a cooperative, supportive, and inclusive approach to problems and to work, as well as a sincere concern for others, work tasks, and the environment.

As Glavas (2016) points out regarding the concept of social responsibility, SS is also a concept that is multi-layered. It includes the micro level (individual), the meso level (e.g., community, work organization, region), and the macro level (society, country). The study of concepts that are inherently multi-layered and transdisciplinary (Lake et al., 2016) risks fundamental errors in social science-errors at the level of data collection, pooling, and analysis, as well as errors in the application of theories that examine a particular concept from a particular level. For this reason, further investigation requires cooperation in science and an expansion of knowledge and skills in psychology. “To more fully understand the psychological nature of working, careful considerations are needed of relevant social, economic, political, and historical forces, which shape, constrain, and facilitate many aspects of contemporary working.” (Duffy et al., 2016, p. 128).

In the introduction, we presented a hypothetical model that helped us define the objectives of the literature review (Figure 1). In it, we defined the reciprocal links between the SDGs of wellbeing and health (SDG-3), gender equality (SDG-5), decent work (SDG-8), safety (SDG-11), and peace (SDG-16) in ensuring the SS of the work environment. The review of the literature indirectly confirmed the interconnectedness of societal, organizational, and individual levels, which together exert an influence on the SS principles and practices of the work environment. Crisis situations such as the COVID-19 pandemic (Trautrims et al., 2020) or the socio-political crisis (Ibrahim, 2021) confirm that security and peace are fundamental starting points for ensuring SS and for achieving the other SDGs identified. At the organizational level, security is also a fundamental feature of SS organizations (Harlin and Berglund, 2021). Although we did not identify many articles that emphasize the role of crisis situations and change management, the identified articles that address the construct of SS and sustainability at the societal (e.g., Trautrims et al., 2020; Ibrahim, 2021) or at the organizational level (Appelbaum et al., 2016; Harlin and Berglund, 2021) emphasize that crisis situations can wear down trust in organizations and society, increase the vulnerability of vulnerable groups (Trautrims et al., 2020; Ibrahim, 2021), and jeopardize the implementation or maintenance of SS principles (Appelbaum et al., 2016; Harlin and Berglund, 2021). The present is therefore an opportune time to reassess efforts to make work and society more sustainable.

In the center of Figure 4 are four circles describing society as the innermost circle, in contrast to the usual psychological approach to describing environmental models for the workplace and personal development in the broader environment and its subsystems. SS is a social construct that must first be established as such in the functioning of the social system as a whole-politics, economics, legislation, labor, welfare, health, and education. Sustainable functioning cannot be achieved at the level of individual organizations and communities, nor by accumulating or increasing the inputs of individuals. Societies in which formal systems and subsystems function according to the principles and values of sustainability are in themselves supportive mechanisms for implementing the goals of SS in communities and organizations. Such societies also include mechanisms that promote pro-social and pro-environmental motives and behaviors in individuals through role models and advocacy. The fundamental ethic of action at the micro, meso, and macro levels in such a society is authentic care (Carmeli et al., 2017; Williams, 2018), which also enables sincere collaboration, participation, and voice for vulnerable groups. The model in Figure 4 therefore underscores the relational nature of the future individual, organizational, and societal development toward SS, in which WOP research and practice play a fundamental role in linking the attitudes, decisions, and behaviors of working people to societal and organizational SS goals and practices. As we predicted in Figure 1, the prerequisite for such an orientation in society and organizations is peace and safety achieved in an uncompromising social contract-a sincere social contract that prioritizes the needs of individual survival, society, and self-determination over political interests (Ibrahim, 2021). The model in Figure 4 has a particularly important implication for policymakers. Changes toward sustainable functioning at the individual and societal levels will not be achieved without changes in societal subsystems, including the educational system in which future generations develop. To achieve changes in the relational and responsibility perspective (from the individual or individualized to the relational perspective), there must also be individual and community characteristics that include, among others, the moral and value dimensions of development.

In conclusion, it should be emphasized that the concept of SS in relation to the WOP is not new. Ultimately, of course, all efforts in this field of psychology are, by basic definition, related to the study, interpretation, and design of the work environment that contributes simultaneously to efficiency, but also to a positive impact on the lives of people, organizations, and society (Blustein et al., 2016). Especially in crisis situations, it is necessary to ensure approaches and attitudes that do not promote stereotypes, prejudice, and discrimination and do not diminish the rights of vulnerable groups. Rather, the crisis can serve as an opportunity to “unlearn” certain entrenched practices and introduce new, more sustainable (Trautrims et al., 2020).

The review of the literature is not exhaustive and is limited by the search terms, the criteria for inclusion and exclusion of scholarly articles, and the databases we used in the search and the accessibility of the works. To some extent, it is also dependent on subjective elements and mechanisms of categorization of constructs. However, we have endeavored to control for this by working in parallel with the two authors. The breadth of the concept of SS requires different approaches and knowledge of the social sciences. For this reason, similar studies in the future should be conducted with an interdisciplinary approach, which would increase the reliability and validity of the conclusions obtained.

## Data availability statement

The raw data supporting the conclusions of this article will be made available by the authors, without undue reservation.

## Author contributions

DKG and KB designed the study, conducted the literature review, analysis, and synthesis of the results. Both authors contributed to the interpretation of the literature review, reviewed it for important intellectual content, and approved it for publication.

## Funding

The publication of the paper was partly financially supported by the Slovenian Research Agency within the research projects Evaluation of the sustainable development of the urban environment through the parameters of social infrastructure and life satisfaction (no. J5-3112) and Psychological and neuroscientific aspects of cognition (research core funding no. P5-0110).

## Conflict of interest

The authors declare that the research was conducted in the absence of any commercial or financial relationships that could be construed as a potential conflict of interest.

## Publisher's note

All claims expressed in this article are solely those of the authors and do not necessarily represent those of their affiliated organizations, or those of the publisher, the editors and the reviewers. Any product that may be evaluated in this article, or claim that may be made by its manufacturer, is not guaranteed or endorsed by the publisher.
